# Memantine as a neuroprotective agent in ischemic stroke: Preclinical and clinical analysis

**DOI:** 10.3389/fnins.2023.1096372

**Published:** 2023-01-19

**Authors:** Diego Pichardo-Rojas, Pavel Salvador Pichardo-Rojas, José Manuel Cornejo-Bravo, Aracely Serrano-Medina

**Affiliations:** ^1^Facultad de Medicina y Psicología, Universidad Autónoma de Baja California, Tijuana, Mexico; ^2^Vivian L. Smith Department of Neurosurgery, The University of Texas Health Science Center at Houston, Houston, TX, United States; ^3^Facultad de Ciencias Químicas e Ingeniería, Universidad Autónoma de Baja California, Tijuana, Mexico

**Keywords:** memantine, acute stroke, ischemia-reperfusion, NMDA, dementia, neuroprotection

## Abstract

The primary mechanism for neuron death after an ischemic stroke is excitotoxic injury. Excessive depolarization leads to NMDA-mediated calcium entry to the neuron and, subsequently, cellular death. Therefore, the inhibition of the NMDA channel has been proposed as a neuroprotective measure in ischemic stroke. The high morbimortality associated with stroke warrants new therapies that can improve the functional prognosis of patients. Memantine is a non-competitive NMDA receptor antagonist which has gained attention as a potential drug for ischemic stroke. Here we analyze the available preclinical and clinical evidence concerning the use of memantine following an ischemic stroke. Preclinical evidence shows inhibition of the excitotoxic cascade, as well as improved outcomes in terms of motor and sensory function with the use of memantine. The available clinical trials of high-dose memantine in patients poststroke have found that it can improve patients’ NIHSS and Barthel index and help patients with poststroke aphasia and intracranial hemorrhage. These results suggest that memantine has a clinically relevant neuroprotective effect; however, small sample sizes and other study shortcomings limit the impact of these findings. Even so, current studies show promising results that should serve as a basis to promote future research to conclusively determine if memantine does improve the outcomes of patients’ post-ischemic stroke. We anticipate that future trials will fill current gaps in knowledge, and these latter results will broaden the therapeutic arsenal for clinicians looking to improve the prognosis of patients poststroke.

## 1. Introduction

Acute stroke is the acute onset of focal neurological findings in a vascular territory due to underlying cerebrovascular disease (Katan and Luft, 2018). Acute stroke remains a significant cause of morbidity and mortality worldwide, currently the second leading cause of death worldwide, accounting for 6–7 million deaths in 2019 (Katan and Luft, 2018; Feigin et al., 2021). Stroke can be classified etiologically into ischemic stroke, which accounts for 62 percent of stroke cases; intracerebral hemorrhage, representing 28 percent; and subarachnoid hemorrhage, representing 10 percent of all global incident cases of stroke (Krishnamurthi et al., 2013; Feigin et al., 2021). Even though both ischemic and hemorrhagic are highly prevalent, ischemic stroke remains far more common, representing up to 87 percent of stroke cases in the U.S. (Roger et al., 2011). The most common consequence of a stroke is neurologic deficits. Stroke is a leading cause of chronic disability worldwide, with up to 30% of stroke survivors becoming permanently disabled. The most common disabilities are hemiparesis, difficulty walking, aphasia, and depression (Roger et al., 2011). Without medical intervention, it is estimated that 62% of stroke patients become dependent or die after 6 months (Heller et al., 2000). Current best medical therapy results in improved functionality, decreased recurrence, complications, and mortality (Phipps and Cronin, 2020). In the last few decades, stroke mortality has been consistently decreasing. According to the CDC, from the year 2000 to 2015, the annual stroke death rate decreased by 47.56% (Centers for Disease Control and Prevention NC for HS, 2016), while the overall burden of disease in that same period, measured by the World Health Organization in Disability Adjusted Life Years (DALYs), just decreased 15.95% (World Health Organization, 2022). An important cause of reduction in morbimortality of stroke has been the advancement of medical therapies available, that while having had a significant benefit on mortality, also result in patients living for longer with disabilities, which is reflected in the minor change in DALYs relative to mortality. The data further emphasizes the drastic consequences of stroke-related disabilities not only in the quality of lives of patients but also in the burden of their caregivers. Hence the importance of striving to keep improving the overall management of patients with stroke.

The gold standard for reduction of mortality and morbidity in ischemic stroke, the most common cause of stroke, is reperfusion therapy, either through thrombolysis or thrombectomy, and despite their benefits, in actual practice, only around 1–3% of stroke patients receive reperfusion therapy (Fisher et al., 2005), and of those who receive it, a majority are still left with disabling neurologic deficits (Grefkes and Fink, 2020). Despite the significant advances made in the treatment of acute stroke over the years, the available therapeutic options for stroke patients are minimal, further emphasizing the need for more research, focusing not only on the acute treatment of stroke but also exploring the recovery options of patients after suffering the acute event. It is crucial to comprehensively analyze any potential therapeutic measure in an unbiased manner to determine potential benefits in the prognosis and functionality of stroke patients. The current search for therapies for ischemic stroke has been based on modifying the natural timeline of events following a stroke. This review aims to examine the currently available evidence supporting the use of one of these potential therapies in ischemic stroke, the drug memantine, presenting evidence supporting its utility and its setbacks. There is solid preclinical evidence supporting the use of memantine for ischemic stroke, yet clinical evidence supporting this drug has been lacking (Seyedsaadat and Kallmes, 2018). In the last few years, there has been a growing amount of evidence supporting the use of memantine in the setting of ischemic stroke, which will be analyzed and presented throughout this review.

## 2. Current stroke treatment

The reduction of mortality in stroke has been partly thanks to the control of risk factors known to increase the risk of death in stroke. The control of isolated systolic hypertension by antihypertensive therapy has been shown in clinical trials to be an essential measure associated with reductions in risk, incidence, and mortality of stroke (Lackland et al., 2014). Atrial fibrillation is also a well-documented risk factor for stroke and systemic embolism; warfarin and oral anticoagulant, including the non-vitamin K antagonist oral anticoagulants (NOAC) as factor Xa inhibitors and direct thrombin inhibitors, are all effective in preventing atrial fibrillation-related stroke; NOAC has been shown to correlate with a significantly lower risk of intracranial hemorrhage than vitamin K antagonist in patients with atrial fibrillation without prior intracranial hemorrhage (Liu et al., 2022). Otherwise, observational studies demonstrated that anticoagulation with vitamin K antagonists correlated with a lower rate of ischemic stroke and no significantly increased intracranial hemorrhage recurrence compared with antiplatelet agents or no antithrombotic medication (Korompoki et al., 2017). The most common side effects caused by the current standard of care are bleeding complications, even more so with vitamin K antagonists than with NOACs (Tsai et al., 2020). Current lines of treatment are based on managing the vascular factors that underlie ischemic stroke. However, there are currently no therapies that act upon the cellular mechanism of neuron death behind ischemic neurotoxicity (Grefkes and Fink, 2020; Matei et al., 2021).

## 3. Role of NMDA in neuron death in ischemic stroke

The current understanding of the cause of neuron death in stroke is due to an excessive excitatory transmission in the setting of ischemia, and one of the main drivers of these events is excitatory cation channels. The NMDA (*N*-methyl-D-aspartate) ionotropic receptor is a major mediator for excitatory transmission in our brain (Rezvani, 2006). It is expressed in 80% of cortical neurons and is involved in many physiological processes, such as memory formation and synaptic long term-potentiation (Conti, 1997). The NMDA receptor can induce metabolic and transcriptional changes in neurons by regulating calcium entry following an excitatory stimulus (Sossa et al., 2006). The NMDA channel becomes permeable to calcium ions when a neuronal depolarization is coupled to glutamate binding in its synapse. The latter phenomenon allows neurons to have a graded response to stimulus, with an initial depolarization mediated by other glutamatergic receptors, such as AMPA. When a neuron is depolarized enough, the NMDA receptor allows for metabotropic changes in a neuron mediated by increased calcium permeability (Kandel et al., 2014).

This receptor can also become pathologically hyperactive when an increased extracellular accumulation of glutamate occurs in ischemic conditions (Zhou et al., 2013). The increased glutamate release, coupled with an inability to repolarize neurons, results in an excessive calcium entry through the hyperactive NMDA channel. The high intracellular calcium concentration results in the activation of various pathways, which results in neuronal death. *In vitro* studies have identified that it is mainly through the NMDA channel that calcium enters the neuron, increases reactive oxygen species (ROS) production, increases mitochondrial membrane permeability, and induces neuron death (Lai et al., 2014). The excessive glutamate release and the increased calcium influx are the main drivers of excitotoxicity, which is thought to be the primary mechanism of neuron death in the early stages of ischemic stroke (Zhou et al., 2013).

## 4. Pathophysiology of ischemic stroke

Understanding the events that follow an ischemic stroke allows us to make sense of the current therapeutic endeavors to treat stroke patients. The occlusion of a cerebral vessel can be of embolic or thrombotic origin. In both cases, the resulting decrease in blood flow will cause dysfunction of normal neuronal activity, followed by ischemic injury and irreversible cell death (Moskowitz et al., 2010). The neurons most proximal to the occluded vessel will die most rapidly, thus forming an ischemic core. At the same time, most distal to the occlusion, there will be an area of electrically and functionally stunned neurons, called the penumbral zone (Şekerdağ et al., 2018). These neurons can follow one of two paths: recover their function through perfusion restoration, or die if the ischemia persists (Moskowitz et al., 2010).

In neurons, the initial decrease in oxygen causes a reduction in ATP concentration and hence a dysfunction of the Na, K-ATPase (Moskowitz et al., 2010). The transmembrane electrical imbalance results in neuronal depolarization, which subsequently impairs the neuronal capacity to transmit action potentials (Campbell et al., 2019). This causes a sizeable excitatory neurotransmitter release (mainly glutamate) in the depolarized ischemic core, which causes a wave of self-propagating electrical activity through the areas surrounding the infarction zone (Moskowitz et al., 2010). The release of glutamate, coupled with neuronal depolarization, results in the opening of AMPA and NMDA cation channels, further worsening the electrolyte imbalance and resulting in excitotoxic cell injury (Pál et al., 2020). The unregulated entry of calcium through the NMDA receptor activates intracellular proteases, endonucleases, and lipases, among other enzymes that trigger apoptotic pathways and result in cell death (Pál et al., 2020). This excitotoxic pathway is the primary mechanism for cell death in the early stages of stroke (Zhou et al., 2013). Ischemia by itself also causes an increased free radical production, which results in cellular necrosis and a disruption of the blood–brain barrier in the ischemic core (Moskowitz et al., 2010). The neurons in the penumbra, which have not undergone cell death, but have ceased electrical activity due to the decreased blood flow, will remain at high risk for irreversible injury (Moskowitz et al., 2010). These neurons initially survived thanks to collateral blood flow, which allowed cells to maintain more than 20 percent of baseline perfusion. Still, the continued hypoperfusion and the subsequent neuroinflammatory response can further hinder their viability (Moskowitz et al., 2010; Campbell et al., 2019).

Microglial activation, which occurs the first hours following ischemia, will create a proinflammatory environment by releasing TNFα and IL1β (Moskowitz et al., 2010; Jayaraj et al., 2019). Other inflammatory cells, such as neutrophils, monocytes, and IL-17-producing lymphocytes, will also migrate and promote debris cleanup (Moskowitz et al., 2010; Jayaraj et al., 2019). In the first 48–96 h after ischemia, astrocytes undergo reactive astrogliosis, becoming hypertrophic and creating a glial scar (Campbell et al., 2019). The coupling of the ongoing neuroinflammatory response, and the antiproliferative effect of reactive astrocytes, will induce the neurons in the penumbral zone to undergo apoptosis and autophagy (Şekerdağ et al., 2018), which will be most pronounced at the 3rd-day day post-ischemia (Angelo et al., 2009). The viability of the penumbral neurons will depend on their environment; in the days following a stroke, angiogenesis will take part in increasing the perfusion of the peri-infarct zone (Adamczak and Hoehn, 2015), and at the same time, there will be a sizeable synaptic rewiring, which will be vital to the process of stroke recovery (Hara, 2015). Current therapies for acute stroke aim to increase the survivability and functionality of the peri-infarct neurons; through reperfusion or experimental methods, such as attenuation of the neuroinflammatory response; and a blockade of proapoptotic second messengers (Liu et al., 2010).

## 5. Blockage of NMDAR as a neuroprotective mechanism

The inhibition of the main pathway of neuronal damage is an attractive target for stroke treatment, but many inhibitors of the excitotoxic cascade have not been successful in clinical trials. Following the occlusion of a vessel, the ischemic changes in brain tissue depend on the degree of hypoxia (Sodaei and Shahmaei, 2020). The site which suffers the most significant decrease in blood flow is the ischemic core, where neurons rapidly undergo cell death (Phan et al., 2002). While in the penumbra, the reduction in blood flow has caused the complete cessation of electrical activity but has not induced the morphological changes of cell death (Phan et al., 2002). The neurons in the penumbral zone will undergo excitotoxic neuron death in the following 4–24 h unless perfusion improves (Li and Wang, 2016; Fifield and Vanderluit, 2020). Despite the attractive idea of inhibiting the excitotoxic cascade through the blockade of the NMDA receptor, there have previously been many clinical trials with NMDAR blockers that have failed to show improvement in the morbidity or mortality of stroke patients. NMDA channel blockers have been demonstrated to be neuroprotective in preclinical models of ischemic stroke. However, these results have not been able to be replicated in a clinical setting (Lai et al., 2014). The reasons for the unsuccessful clinical transition of these NMDAR antagonists are unclear. However, proposed reasons include, but are not limited to, inappropriate dosage for neuroprotection, intolerable side effects, administration out of their neuroprotective window, poor experimental design, and variable patient populations, among others (Lai et al., 2014). The pharmacodynamic differences between NMDAR antagonists are partly responsible for the varying results in clinical effectiveness, highlighting the importance of both efficacy and tolerability.

## 6. Memantine

Memantine (3,5-dimethyltricyclo[3.3.1.1^3,7^]decan-1amine or 3,5-dimethyladamantan-1-amine) is a primary aliphatic amine. It is a member of the adamantanes, in the same class as amantadine (Lee et al., 2016). Memantine is an *N*-methyl-D-aspartate (NMDA) receptor antagonist. The FDA approved memantine in 2003 for its use in moderate to severe Alzheimer’s disease. Continuous activation of the NMDA receptors in the central nervous system caused by glutamate is thought to be partially responsible for the symptoms of Alzheimer’s disease. The pharmacological effect of memantine occurs via its activity as a non-competitive (open-channel) rapid off-rate NMDA receptor antagonist, which prevents the action of glutamate on its receptor. Memantine preferentially binds hyperactive NMDA receptors without disrupting the normally functioning NMDA cation channels (Chen and Lipton, 2006). This property allows it to act mainly in pathologically depolarized brain regions that inhibit calcium influx into cells, normally caused by chronic NMDA receptor activation by glutamate. Despite these antagonist effects, memantine has not been proven to prevent or delay neurodegeneration in patients diagnosed with Alzheimer’s (Robinson et al., 2006). Due to the unique kinetics of memantine, it has excellent clinical tolerability, in contrast to other high-affinity NMDA channel blockers (Chen and Lipton, 2006).

The interest in memantine for vascular diseases follows many years of its use for dementia. Memantine has been shown to improve behavioral disturbances (decreased aggression and liability), delays cognitive decline, and improve overall mood when given at a dose of 20 mg a day as monotherapy, or alongside a Cholinesterase Inhibitor (rivastigmine, galantamine, or donepezil), in patients with moderate to severe Alzheimer’s disease (Wilcock et al., 2008; Kishi et al., 2017; McShane et al., 2019). Adverse effects are rare, with a low rate of dizziness and headaches. Despite this, there are no differences between rates of treatment discontinuation between memantine and placebo groups (Kishi et al., 2017; McShane et al., 2019). There is a lack of evidence to support the use of memantine for mild Alzheimer’s disease, with reviews showing that it provides no benefits in this group of patients (Schneider et al., 2011). The mechanism through which memantine improves symptoms in dementia is not fully understood, with current evidence supporting that NMDA blockade decreases the progressive death of cholinergic neurons. Through the reduction of oxidative stress, there is a decrease in the expression of amyloid precursor protein and tau proteins (Rogawski and Wenk, 2003; Dominguez et al., 2011). Evidence supports the use of memantine in other neurodegenerative diseases, such as vascular dementia, in which the NMDA blockade in the setting of chronic cerebrovascular disease improves cognitive function. The neuroprotective effect of memantine in the setting of ischemia encouraged research in other vascular diseases, such as stroke.

Memantine at high doses can reduce neuronal synaptic plasticity, which is involved in learning and memory processes. At lower concentrations, typically used in the clinical setting, memantine can enhance neuronal synaptic plasticity in the brain, improve memory, and act as a neuroprotectant against the destruction of neurons caused by excitatory neurotransmitters (Rogawski and Wenk, 2003). Memantine has a minimal activity for voltage-dependent K^+^, Ca^2+^, and Na^+^ channels, benzodiazepines, dopamine, adrenergic, histamine, GABA, and glycine receptors. This drug has shown antagonist activity at the serotonin 5HT_3_ receptors. Memantine does not affect the reversible acetylcholinesterase inhibition normally caused by tacrine, galantamine, or donepezil (Kishi et al., 2017).

Memantine has a bioavailability close to 100%, reaching *C*_*max*_ within 3–8 h and a half-life between 60 and 70 h. Memantine and its metabolites are mainly excreted via the kidneys, contributing to tubular secretion. About 80% of the circulating memantine dose is present in humans as the parent compound. Memantine undergoes hydroxylation and oxidation, but CYP does not catalyze these reactions, hence a low risk for drug interactions (Noetzli and Eap, 2013).

## 7. Preclinical evidence of neuroprotection by memantine

**FIGURE 1:**
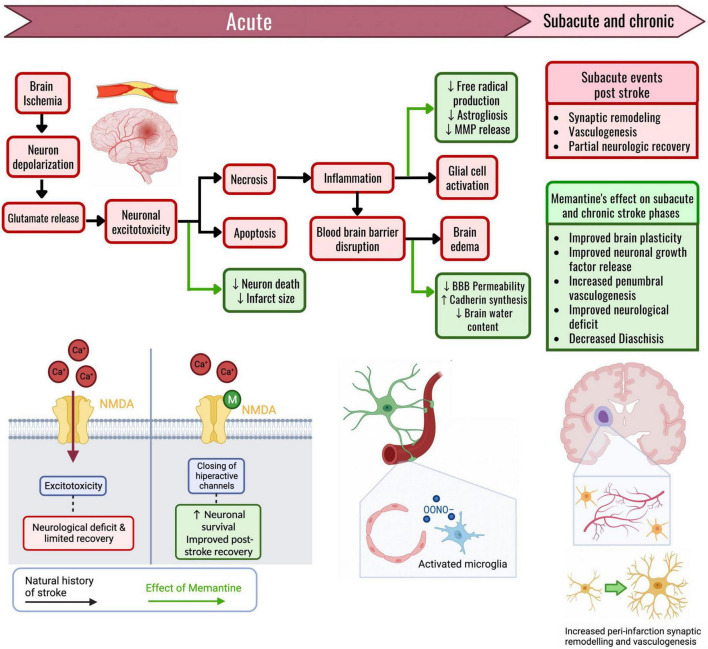
The effects of memantine on the pathophysiologic cascade of an ischemic stroke. The effects of memantine administration are marked through green arrows. When a region of the brain suffers ischemia, the neurons initially depolarize. The wave of depolarizations triggers glutamate release, and neuronal permeability to calcium increases due to the NMDA channel opening. Prolonged ischemia with increased intracellular calcium thus causes excitotoxicity, with cellular death through necrosis and apoptosis. The main effect of memantine is through the blockade of the hyperactive NMDA channels that drive excitotoxicity. Memantine given in these acute stages of ischemic stroke results in an inhibition of neuron death and causes a reduction in stroke size. Tissue necrosis triggers inflammation, disrupts the blood–brain barrier (BBB) through endothelial damage, and activates both microglia and astrocytes, causing brain edema. Memantine can reduce the activation of reactive cells, thus reducing peroxynitrite formation and MMP release, consequently helping maintain the impermeability of the BBB. The preservation of BBB permeability results in an attenuated brain edema formation. Following a stroke, in the subacute and chronic stages, there is a partial functional recovery mainly due to synaptic remodeling and peri-infarct vasculogenesis. If memantine is given during these latter stages, there is an increase in the concentration of neuronal growth factors, along with an improved capacity for synaptic remodeling. Memantine induces an increase in axonal sprouting, and an increase in peri-infarct vasculogenesis, thus giving further support to the recovering neurons around the ischemic core. Through these mechanisms, memantine improves the functional recovery that follows an ischemic stroke.

Preclinical studies have shown that memantine post-stroke can decrease infarction size, increase peri-ischemic vascularity, inhibit neuronal apoptosis in the penumbral zone, decrease brain edema formation, and improve post-ischemic neurological function (Figure 1; Görgülü et al., 2000; Culmsee et al., 2004; Chen et al., 2017). Memantine has been proven to provide post-ischemic neuroprotection via multiple mechanisms, including inhibition of apoptosis (Tuo et al., 2021), NMDA inhibition mediated excitotoxicity (Wu and Tymianski, 2018), preserving intracellular ATP stores (Tuo et al., 2021), and increasing tissue concentration of neuron-specific growth factors (Wang et al., 2017). The magnitude of the benefit of memantine administration post-ischemia depends on how early it is administered (Seyedsaadat and Kallmes, 2018). It has also been shown that memantine can decrease the neuronal death caused by reperfusion injury; during *in vitro* studies and a preclinical model when co-administered with Recombinant Tissue Plasminogen Activator (rtPA) (Montagne et al., 2012; Liu et al., 2018). The preclinical models of the effects of memantine on ischemic stroke are mainly murine and *in vitro* cell cultures, as summarized in Supplementary Table 1.

This review mainly focuses on the effects of memantine administered post-stroke. Still, various *in vitro* trials have given us a more in-depth look into the molecular mechanisms of neuroprotection. *In vitro* models of ischemia have identified a dose-dependent neuroprotective effect following ischemia (Chen et al., 2017), with no neuroprotection at low dosages (memantine at 0.1 μM); and a significant attenuation of cell death following hypoxia at higher concentrations (memantine at 10 and 50 μM) (Seif el Nasr et al., 1990). The neuroprotective effects of memantine in hypoxia models are augmented when coupled with other therapies, such as memantine with an *in vitro* model of hypothermia (Landucci et al., 2018).

### 7.1. Memantine post-ischemic use

Culmsee et al. (2004), in a mouse model, showed that administering 20 mg/kg of memantine 5 min after stroke reduced cortical infarction size by 10%, an effect that did not carry over when administering memantine 30 min after stroke (Culmsee et al., 2004). Even so, there is evidence that memantine can decrease infarction volume when coupled with reperfusion (Kilic et al., 2013). The administration of memantine can protect against the earliest effects of NMDA-mediated excitotoxic neuronal death; this effect, however, has repeatedly failed to carry over when administering memantine 2 h after the onset of ischemia. The benefits after this time frame occur mainly in motor, sensory, and behavioral function (Seyedsaadat and Kallmes, 2018).

López-Valdés et al. (2014), showed that the administration of memantine 2 h after stroke in a mice model for 28 days did not change the volume of infarction but significantly increased forepaw sensory perception (through sensory brain mapping) and motor function (through motor cylinder test) when compared to a control. There was also an increase in peri-infarct vascularity and decreased reactive astrogliosis at 28 days post-stroke (López-Valdés et al., 2014), the latter of which, studies have suggested, can reduce inflammation and promote functional recovery (Shen et al., 2021). Other studies have found that starting memantine 2 h after a stroke can result in a more significant improvement of neurological function at 72 h post-stroke (Aluclu et al., 2014). At this time frame, memantine use also provides a considerable mortality benefit (Kalemenev et al., 2012). It is well established that following the initial deficits in a stroke, patients tend to improve during the first few months, resulting in a partial recovery (Grefkes and Fink, 2020). When comparing neurological improvement post-stroke, it is noticeable that the initiation of memantine, 3 h after stroke, has been shown to enhance motor function when evaluated at 24 h post-stroke while decreasing anxious behavior on the 7th day after ischemia (Babu and Ramanathan, 2009).

In addition, a study by Wang et al. (2017), it was demonstrated that the use of memantine at 20 mg/kg/day for 28 days, starting at 72 h after stroke, resulted in an improvement in motor coordination (through Rotarod and tight rope tests), an increase in peri-infarct vascularity, and decreased astrogliosis in mice (Wang et al., 2017). The reduction of astrogliosis and other results that show a reduced edema formation, demonstrated that memantine attenuates the inflammatory response to stroke (Kilic et al., 2013). It was also shown that the administration of memantine resulted in fibers from the contralesional corticospinal tract sprouting and decussating toward various ipsilesional motor nuclei, showing improved post-stroke brain plasticity. Functional motor improvement post-stroke has been shown to occur partly thanks to the formation of new tracts and synapses from neurons related to the infarcted territory (Grefkes and Fink, 2020). In this study, memantine, beginning at 72 h, did not decrease the cortical volume of infarction but reduced the striatum’s secondary atrophy (Wang et al., 2017). Kim et al. (2021), also reported that memantine did not reduce the size of primary infarction but reduced secondary atrophy of the ipsilesional thalamus. The secondary atrophy of a site, following its disconnection from a territory that has suffered ischemia, is termed “diaschisis” (Zhang et al., 2012). Secondary neurodegeneration has been associated with limited recovery and the worst outcomes post-stroke (Ni et al., 1998). The beneficial effects of memantine administration are most significant when administered early, the neuroprotective effect decreases as time goes on, yet there is still a significant benefit if memantine is begun even at 72 h post-stroke (Seyedsaadat and Kallmes, 2018).

### 7.2. Memantine on brain edema

The loss of integrity of the blood–brain barrier (BBB), and the formation of brain edema from cytotoxic and vasogenic sources, are critical pathologic events in stroke (Michinaga and Koyama, 2015). The cerebral edema is initially cytotoxic due to transmembrane ion imbalance of Sodium and Calcium. Still, after 4–6 h, there is an increase in the permeability of the BBB, particularly in the areas around the ischemic core. A loss of tight junction proteins causes an increase in permeability, membrane damage by free radicals, and enzymatic digestion of barrier proteins (particularly the MMP family of enzymes), among others.

Memantine has been shown to reduce the formation of brain edema and decrease the levels of inflammatory mediators that increase BBB permeability (Görgülü et al., 2000). The earliest evidence of how memantine can modify the development of edema in stroke was developed by Görgülü et al. (2000). They showed that if memantine was administered 15 min after the onset of ischemia, the rat models developed less edema at the infarct periphery compared to control (measured with cerebral water content), while also decreasing BBB permeability (Görgülü et al., 2000). The decreased peri-infarct edema was also coupled with a decreased infarct volume and decreased post-stroke neurologic deficit. Kilic et al. (2013), also demonstrated that memantine decreased BBB permeability 90 min after ischemic stroke when administered alongside melatonin. By itself, memantine also decreases DNA fragmentation, and neuroinflammatory response proteins at the ischemic core; p38, MAPK, and p21 (Kilic et al., 2013). *In vitro* trials on brain endothelial cells have also shown that following ischemia, memantine helps maintain the impermeability of the endothelial monolayer, mainly through the downregulation of proinflammatory cytokines (IL-1β and TNFα) and by upregulation of the KLF2 transcription factor, which maintains the integrity of BBB through increased synthesis of occludin proteins between endothelial cells (Liu et al., 2018). Following ischemia, the release of Matrix metalloproteinases (MMPs) can acutely increase BBB permeability; Memantine has been found to decrease the amounts of MMP2 and MMP9 (Chen et al., 2016; Liu et al., 2018), preventing the breakdown of BBB collagen fibers, and also decreasing the activation of microglia. Other models of acquired brain injury have also found that memantine can ameliorate the development of cerebral edema through decreased peroxynitrite formation, increased occludin proteins, and decreased inflammatory cytokines (Chen et al., 2021).

### 7.3. Memantine on neurotrophic actors

A growing topic of stroke research are neurotrophic factors, and their effects on synaptic plasticity and post-stroke rehabilitation. Neurotrophins, such as BDNF (brain derived neurotrophic factor), NGF (nerve growth factor), GDNF (glial cell line-derived neurotrophic factor), among others, are responsible for synapse maturation, preservation of normal cognitive function, neurite arborization, and overall neuronal maintenance (Liu et al., 2020). Following a stroke, neurotrophins tend to decrease, in a manner correlated with stroke severity (Chaturvedi et al., 2020). The role of BDNF post-stroke has been of particular interest, having been shown to attenuate stroke-induced apoptosis, improve synaptic remodeling, stimulate neurogenesis, and improve post-stroke sensorimotor recovery (Schäbitz et al., 2007; Liu et al., 2020). Considering all of this, it is very interesting to analyze how memantine has been shown to increase BDNF in the area surrounding an infarct zone (Martínez-Coria et al., 2021); the rise in neurotrophins occurring both in ipsilesional and contralesional zones of the brain (Wang et al., 2017). These findings suggest that the neuroprotective properties of memantine and its effects on post-stroke functional recovery are due, in part, to increases in the endogenous synthesis of neurotrophic factors.

### 7.4. Memantine on reperfusion injury

Reperfusion injury is an essential mechanism of neuron death in both the natural history of stroke and after therapeutic reperfusion (Nour et al., 2013). An *in vitro* study by Liu et al. (2018), demonstrated that the use of memantine in a model of reperfusion results in an inhibition of the release of cytotoxic cytokines (IL-1b and TNF-a) and, at the same time, reducing both endothelial permeability and, expression of matrix metalloproteinases (Liu et al., 2018). These enzymes are involved in the neuroinflammatory response after stroke (Rosell et al., 2005). The coupling of memantine with reperfusion has been shown to decrease infarct size, improve neurological function, and improve recovery at 1 week (Aluclu et al., 2014). It has also been shown to improve safety and reduce neuronal death when co-administered with rtPA up to 4 h after stroke (Montagne et al., 2012). Reperfusion injury is an inevitable consequence of therapeutic revascularization (Cowled and Fitridge, 2011), but these results open the possibility of memantine as an adjunct drug to help ameliorate neuron injury when used along rtPA.

### 7.5. Memantine effects at distinct dosages

The pharmacologic effects of memantine vary between dosages and the time frame of the neurological insult in which it is administered. The unique kinetic properties of memantine result in the predominant blockade of pathologically overactive NMDA channels (Lipton, 2006). Despite the preclinical and *in vitro* evidence supporting its neuroprotective effect at dosages between 20 and 30 mg/kg/day following an ischemic event, it should be noted that this dosage has also been documented to be neurotoxic in distinct conditions. Trotman et al. (2015), described the dose-dependent effects of memantine when given before an ischemic event, showing that low-dose memantine (0.2 mg/kg/day) can significantly reduce stroke volume and improve behavior scores, while on the contrary, if memantine is administered at high dose (20 mg/kg) for 24 h before ischemia, the size of ischemic injury is increased (Trotman et al., 2015). These findings are consistent with Creeley et al. (2006), who described how administering early memantine high dose can result in neurological functional deficits (Creeley et al., 2006). Current evidence suggests that the high-dose neurotoxic effects are not NMDA mediated but could be due to the interaction of memantine with other channels, such as serotonin 5-HT receptors (Reiser and Koch, 1989; Trotman et al., 2015). This evidence supports the idea that the inhibitory effect of memantine is potentiated when administered for more extended periods and how this effect can be deleterious at higher dosages (Bakiri et al., 2008). The non-competitive binding to NMDA channels makes it so high doses provide significant benefits in pathological conditions while also having the potential to be neurotoxic in a dose-dependent manner (Trotman et al., 2015).

## 8. Memantine’s clinical use in stroke?

Even though little evidence is available in the literature about the impact of memantine and its specific use on stroke, some preliminary studies have started to illuminate its potential use. It is not the first time memantine has demonstrated some efficacy in vascular-related brain diseases, such as Vascular Dementia. Since memantine has been approved for moderate to severe cases of Alzheimer’s, many patients with Vascular Dementia have been treated with memantine, mainly because Alzheimer’s Disease cannot be ruled out and often because both conditions are comorbid (Kapasi et al., 2017). Up to date, two studies have compared memantine 20 mg/day vs. placebo in patients with mild to moderate Vascular Dementia during 28 weeks (Orgogozo et al., 2002; Wilcock et al., 2002). Results showed improved performance on cognitive scales but not on functional outcomes, such as activities of daily living (Kavirajan and Schneider, 2007; McShane et al., 2019).

Even though Vascular Dementia and stroke are both vascular diseases, their natural history of disease is different. This difference brings up the question: ¿Can memantine have any clinical benefit in patients with stroke?

Regarding patients with ischemic stroke, a small clinical trial explored the short-term outcomes of 53 patients with mild-to-moderate ischemic stroke. The control arm (29 patients) was treated based on the standard care guidelines by the American Heart Association and American Stroke Association (AHA/ASA) and compared with patients who were additionally treated with high-dose memantine (20 mg/kg three times a day) in the first 24 h after ischemic stroke disease onset, and for the following 5 days (24 patients). The outcome was measured by comparing both groups’ changes in the National Institute of Health Stroke Scale (NIHSS). The memantine-treated group showed a significant improvement in the NIHSS (*p* = <0.05), hence suggesting a possible improvement in neurologic function (Kafi et al., 2014). An additional clinical trial done in 2020, that showed similar results has yet to be published (Clinical Trials.gov Identifier: NCT02535611), while another trial of memantine on stroke recovery is currently ongoing (ClinicalTrials.gov Identifier: NCT02144584). Furthermore, another interesting clinical trial also studied 77 patients with mild-to-moderate ischemic stroke and randomized into the intervention (24 patients) or control group (29 patients). Both groups were treated with standard care; the intervention group was additionally treated with 20 mg memantine every 8 h for 5 days and 20 mg once daily for the following 3 months. The measured outcomes were estimated with serum concentrations of neuronal damage biomarkers, matrix metalloproteinases (MMP)-2 and MMP 9; neurologic function was evaluated with the NIHSS and Barthel Index (BI). Five days after the intervention, results showed a significantly smaller increase in serum MMP-9 in the intervention group (*p* = <0.05), but not in the MMP-2 (*p* = >0.05). The memantine group also showed significant clinical improvement, based on NIHSS (*p* = <0.05) and BI (*p* = <0.05) during inpatient hospital care and the following days (Beladi Moghadam et al., 2021). These findings suggest that memantine may improve neurologic function and reduce brain damage by working as a neuroprotective drug.

Aphasia is a loss of the capacity to produce or understand language; cerebrovascular diseases most commonly cause it, and it is also one of the most dreaded consequences of cerebral infarction. Aphasia is a common complication of ischemic strokes, representing up to 15–38 percent of said complications (Wade et al., 1986; Pedersen et al., 1995; Berthier, 2005; Inatomi et al., 2008). Over the years, an effort has been made to target language therapy techniques that help the recovery of patients with aphasia, including constraint-induced aphasia therapy (CIAT, a high-intensity therapy that mainly focuses on restricting non-verbal communication) (Cherney et al., 2008; Szaflarski et al., 2008). In a study of 27 patients with chronic post-stroke aphasia, CIAT was beneficial when used alone and combined with memantine 10 mg twice daily. The benefit of memantine was enhanced when combined with CIAT (Berthier et al., 2009).

Unfortunately, the evidence of the use of memantine in stroke complications and recovery is minimal. Even though many questions are yet to be answered, more research is needed to provide conclusive evidence. There is currently one ongoing clinical trial exploring if memantine can enhance stroke recovery (Clinical Trials.gov Identifier: NCT02144584).

On the other hand, in an alternative randomized, double-blind clinical trial, they compared the use of Memantine and Placebo on the clinical outcome of 64 patients with Intracranial Hemorrhage (ICH). This study assessed several neurological functional scales on admission, on the seventh day, upon discharge, and 3 months after the ICH onset; this was achieved by measuring the NIHSS, BI, modified Rankin scale (mRs), and Glasgow Coma Scale (GCS). The memantine group was treated with 10 mg/day in the first month and 20 mg/day in the second and third months. Results showed a significant increase of the mean in the BI and a decrease in the mRs in the memantine-treated group compared to placebo; these results were measured from admission time until the 3 months (*P* = <0.05). No significant differences were demonstrated when analyzing mortality rate, GCS, or NIHSS score (*P* = >0.05) (Bakhshayesh-Eghbali et al., 2015). This further increases the potential therapeutic landscape suggesting that the benefits of memantine may also extend to other etiological types of stroke; however, further research needs to be done before any conclusions can be drawn. Even though fascinating clinical evidence exists for memantine in vascular-related diseases, evidence still needs to be compelling. Further research needs to be done to discover if memantine can provide definitive clinical benefits for patients in the future.

## 9. Discussion

A solid amount of preclinical evidence backs up memantine as a neuroprotective agent in the setting of ischemic stroke. However, these results have not been directly replicated in a clinical setting. There have been few but significant clinical trials showing that memantine can improve the prognosis of patients post-stroke, but these trials have been limited by their small sample size. As an inhibitor of the excitotoxic cascade, memantine has been found to have excellent tolerability compared to other NMDA channel blockers, with only 2% of patients having significant side effects compared to placebo at the standard dose of 20 mg/day (Alva and Cummings, 2008). Results from clinical trials using high-dose memantine (60 mg/day) in the setting of mild to moderate stroke (NIHSS <17) show a significant benefit in the neurological function and measures of independence of patients post-stroke. The most common side effects at these dosages were nausea, with a 10–25% prevalence in intervention groups (Kafi et al., 2014; Beladi Moghadam et al., 2021). These studies support high-dose memantine as being safe and tolerable. While promising, the utility of memantine as a neuroprotective agent in conditions other than Alzheimer’s disease still requires further research. More extensive clinical trials using the appropriate and effective dosages are needed before determining if memantine improves the outcomes of patients following an ischemic stroke.

## 10. Conclusion

Stroke is a main cause of morbimortality worldwide, and research into therapies that can improve functionality and independence post-stroke is of the utmost importance. The death of neurons in an ischemic stroke is mainly driven by excitotoxicity, resulting in an ischemic core and a penumbral zone with viable neurons. The inhibition of the excitotoxic cascade through a blockade of the NMDA channel can be achieved using memantine. This drug is a well-studied neuroprotective agent for Alzheimer’s disease. The use of memantine as a therapeutic agent in ischemic stroke has been studied in various preclinical models and has shown consistent benefits. The sooner memantine was administered, the greater the benefit in preclinical experiments. On the other hand, the clinical evidence on the use of memantine in ischemic stroke is scarce but significant, showing an improvement in NIHSS and BI in patients post-stroke. Current evidence should serve as the basis for future large-scale studies to investigate if memantine indeed improves the neurological function of patients post-stroke while determining the appropriate dosage, administration route, treatment duration, and the time window in which it could be effective.

## Author contributions

DP-R: conceptualization and writing—original draft preparation. PP-R and JC-B: validation and formal analysis. AS-M and JC-B: writing—review and editing. All authors read and agreed to the published version of the manuscript.
