# Supplementary material for: Memantine as a neuroprotective agent in ischemic stroke: Preclinical and clinical analysis
Source: Front Neurosci. 2023 Jan 19;17:1096372. doi: 10.3389/fnins.2023.1096372 (PMC9893121; doi:10.3389/fnins.2023.1096372)
Supplement: Supplementary file 1 [file Table_1.DOCX]

**Supplementary Table 1.** Summary of preclinical outcomes of memantine in stroke models.

| Reference | Trial | Model | Memantine  Admin. | Condition | Therapy | Results |
| --- | --- | --- | --- | --- | --- | --- |
| Culmsee et al., 2004 | Memantine and clenbuterol on ischemic stroke size | Mice | IP | Permanent MCAO | Memantine 20mg/kg  5 min after ischemia | 10% reduction in stroke size |
|  |  |  |  |  | Memantine 20mg/kg  30 min after ischemia | No significant  size reduction |
|  |  |  |  |  | Memantine 20mg/kg 90 min after ischemia | No significant  size reduction |
| Chen et al., 2017 | Memantine on stroke size, post-stroke functionality, and quantification of apoptotic proteins in the penumbra. | Sprague Dawley rats | IP | MCAO and reperfusion after 1 hour | Memantine 20mg/kg immediately after ischemia. Maintenance dose 12mg/kg every 12 hours | Reduction in stroke size |
|  |  |  |  |  |  | Improved neurological function at 24 hours |
|  |  |  |  |  |  | Decreased concentration of calpain 1 & 2 in the penumbra |
|  |  |  |  |  |  | Increased concentration of PSD95 & MAP2 in penumbra |
|  | Memantine on ATP-depleted neurons | Hippocampal neurons from 18-d rat embryos | Cell culture | Glucose deprivation & ATP depletion for 20 minutes, | Memantine at 1 μM after 20 minutes of ATP depletion | No change in cell death of ATP depleted neurons |
|  |  |  |  |  | Memantine at 10 μM after 20 minutes of ATP depletion | Decreased cell death of ATP-deplete neurons |
|  |  |  |  |  | Memantine at 50 μM after 20 minutes of ATP depletion | Decreased cell death of ATP-depleted neurons |
| Görgülu et al., 2000 | Memantine & MK-801 on stroke size, neurological function, and brain edema formation in the setting of ischemia and reperfusion. | Sprague Dawley rats | IP | MCAO for 2 hours, and then 2 hours of reperfusion | Memantine 10 mg/kg after 15 minutes of ischemia. | Reduction in stroke size |
|  |  |  |  |  |  | Decreased neurological deficit |
|  |  |  |  |  |  | Decreased brain edema |
|  |  |  |  |  |  | Decreased permeability of the BBB at the penumbra |
|  |  |  |  |  |  | No change of BBB permeability at the ischemic core |
| Wang et al., 2017 | Post-acute delivery of memantine on peri-infarct tissue remodeling, contralesional brain plasticity, neurological function | C57BL6/j Mice | SQ | MCAO for 40 minutes, followed by reperfusion | Memantine 4 mg/kg started 72 h after ischemia for 28 days. | No differences in control |
|  |  |  |  |  | Memantine 20 mg/kg started 72 h after ischemia for 28 days. | Improvement in coordination deficits (12 dpt) |
|  |  |  |  |  |  | Increased striatal volume |
|  |  |  |  |  |  | Decreased astrogliosis (49dpt) |
|  |  |  |  |  |  | Increased capillary density (49dpt) |
|  |  |  |  |  |  | Increased concentrations of BDNF, GDNF, VEGF, in ipsi and contralesional striatum and cortex (28 dpt) |
|  |  |  |  |  |  | Increased contralesional pyramidal tract crossover |
| Liu et al., 2018 | Memantine on ischemia-reperfusion | Human Brain Microvascular Endothelial Cells monolayer | Cell culture | Oxygen-glucose deprivation, followed by 21% Oxygen concentration | Memantine at 10 μM in normoxic conditions | Decreased production of IL-1β & TNFα |
|  |  |  |  |  |  | Attenuated permeability increases following ischemia |
|  |  |  |  |  |  | Increased occludin and cadherin protein synthesis |
|  |  |  |  |  |  | Decreased MMP2 release |
|  |  |  |  |  |  | No change in MMP9 release |
| Montagne et al., 2012 | Effect of memantine on reperfusion injury following late thrombolysis | Male Swiss Mice | IV | MCAO for 4 hours, then reperfusion via rtPA | Memantine 20 mg/kg along with reperfusion via rtPA | Decreased stroke size increased the following reperfusion |
|  |  |  |  |  |  | Improved sensorimotor function compared to just reperfusion |
| Kilic et al., 2013 | Memantine and Melatonin on ischemic brain injury, and BBB permeability pathways. | C57BL/6j Mice | IP | MCAO for 90 minutes, followed by reperfusion | Memantine 20 mg/kg immediately after reperfusion. Half dose at 20 min | 50% stroke size reduction |
|  |  |  |  |  |  | Decreased DNA fragmentation in cortex & striatum |
|  |  |  |  |  |  | Decreased IgG BBB extravasation |
|  |  |  |  |  | Melatonin 4mg/kg, Memantine 20 mg/kg immediately after reperfusion.  Half dose at 20 min. | 70% stroke size reduction |
|  |  |  |  |  |  | Decreased DNA fragmentation in cortex & striatum |
|  |  |  |  |  |  | Decreased IgG BBB extravasation |
| López-Valdés et al., 2014 | Memantine on functional stroke recovery | C57B1/6J Mice | Oral | Photothrombosis induced stroke | Memantine 30 mg/kg/day, beginning 2 h after photothrombosis, then continued for 28 days. | No change in stroke size |
|  |  |  |  |  |  | Improved forepaw motor and sensory function |
|  |  |  |  |  |  | Decreased peri-infarct astrogliosis |
|  |  |  |  |  |  | Increased peri-infarct vascularity |
|  |  |  |  |  |  | Increased peri-infarct BDNF |
|  |  |  |  |  |  | No change in peri-infarct GDNF/VEGF |
| Aluclu et al., 2008 | Memantine on ischemic stroke volume and recovery. | Sprague Dawley Rats | NG | MCAO for 2 hours, then reperfusion | Memantine 30mg/kg 2 h after ischemia | Improved neurological function after 24 & 72 hours |
|  |  |  |  |  |  | Decreased infarct size (cerebrum, cerebellum, brain stem) |
| Kalemenev et al., 2012 | Memantine on ischemic stroke outcomes | Wistar Rats | IP | Permanent MCAO | Memantine 10 mg/kg 2 h after ischemia | Prominent decrease in mortality |
|  |  |  |  |  |  | Increased motor activity & exploratory behaviors |
| Babu et al., 2009 | The neuroprotective function of Memantine post-stroke | Sprague Dawley Rats | IP | MCAO for 2 hours, then reperfusion | Memantine 20 mg/kg 1 h after MCAO | Improved neurological recovery at 24 hours |
|  |  |  |  |  |  | Decreased neuronal damage |
|  |  |  |  |  |  | Decreased Lactate, glutamine synthase, glutamate at 72 hours |
|  |  |  |  |  |  | Increased NaK ATPase activity |
|  |  |  |  |  | Memantine 20mg/kg 5 h after MCAO | Improved neurological recovery at 24 hours |
|  |  |  |  |  |  | Decreased neuronal damage |
|  |  |  |  |  |  | Decreased Lactate at 72 hours |
| Kim et al., 2021 | Memantine treatment on the secondary injury of the thalamus | C57BL/6 Mice | IP | Permanent distal MCAO. | Memantine 100 mg/kg, at 4 hours post-stroke, then memantine 50 mg/kg 24 hours post-stroke | Decreased thalamic gliosis at 14 days post-stroke |
| Seif-el-Nasr et al., 1990 | The dose-dependent neuroprotective effect of memantine on hypoxia | Cultured neurons  from the telencephalon of 7-day old chick embryos | Cell culture | Oxygen-glucose deprivation for 30 minutes | Memantine at 0.1 μM for 24 hours, starting after 30 min of glucose deprivation | No attenuation of neuronal damage |
|  |  |  |  |  | Memantine at 1 μM for 24 hours, starting after 30 min of glucose deprivation | Decreased neuronal damage |
|  |  |  |  |  | Memantine at 10 μM for 24 hours, starting after 30 min of glucose deprivation | Decreased neuronal damage |
| Landucci et al., 2018 | Neuroprotective effect of memantine coupled with hypothermia in hypoxic injury | Hippocampal slices of 7 to 9-day old Wistar rats | Cell culture | 30 minutes of oxygen deprivation | Memantine at 1 μM, for 24 hours, after 30 min of oxygen deprivation | No statistically significant decrease in cell death |
|  |  |  |  |  | Memantine at 10 μM, for 24 hours, after 30 min of oxygen deprivation | 50% attenuated neuronal damage |
|  |  |  |  |  | Memantine at 30 μM, for 24 hours, after 30 min of oxygen deprivation | 60% attenuated neuronal damage |
|  |  |  |  |  | Memantine at 30 μM, and 32º hypothermia, for 24 hours, after 30 min of oxygen deprivation | 75% attenuated neuronal damage |
|  |  | 7-day old Wistar rats | IP | Left common carotid artery ligation, followed by exposure to hypoxia for 2 hours | Memantine at 20 mg/kg immediately after hypoxia and repeated dose 2 hours after hypoxia | Statistically significant decrease in infarction size |
|  |  |  |  |  | Memantine at 20 mg/kg, and 4 hours of 32º hypothermia, immediately after hypoxia, and repeated memantine dose 2 hours after hypoxia | Greater reduction in infarct size than memantine by itself |
| Chen et al., 2016 | Memantine pretreatment on neurovascular unit protection mechanisms | C57 BL6 mice | IP | Permanent MCAO | Memantine 20 mg/kg, 30 minutes before MCAO. | Reduced microglia activation and astrocyte damage at 24 hours post-ischemia |
|  |  |  |  |  |  | Reduction in MMP-9 secretion |
|  |  |  |  |  |  | Increased quantity of BBB type IV Collagen |
|  |  |  |  |  |  | Improved BBB integrity post-stroke |
|  |  |  |  |  |  | Higher concentrations of PSD 95. |
| Trotman et al., 2015 | Different dosages of memantine pretreatment in ischemic stroke | C57 BL6 mice | SQ | MCAO for 60 minutes, followed by reperfusion | Mem 0.2 mg/kg every 24 h beginning 24 h pre-stroke | Reduction in corticostriatal lesion size. |
|  |  |  |  |  | Mem 10 mg/kg every 24 h beginning 24 h pre-stroke | No reduction in corticostriatal lesion size |
|  |  |  |  |  | Mem 20 mg/kg every 24 h beginning 24 h pre-stroke | Increase in mean stroke lesion size. |

IP: Intraperitoneal; SQ: Subcutaneous; IV: Intravenous; Mem: Memantine; Admin: Administration; NG: Nasogastric; MCAO: Middle Cerebral Artery Occlusion; PSD95: Postsynaptic density protein 95; MAP 2: Microtubule Associated Protein 2; BBB: Blood Brain Barrier; Dpt: Days post-treatment; rtPA: Recombinant tissue Plasminogen Activator
